# Target Population of Environments for Wheat Breeding in India: Definition, Prediction and Genetic Gains

**DOI:** 10.3389/fpls.2021.638520

**Published:** 2021-05-24

**Authors:** Leonardo Abdiel Crespo-Herrera, Jose Crossa, Julio Huerta-Espino, Suchismita Mondal, Govindan Velu, Philomin Juliana, Mateo Vargas, Paulino Pérez-Rodríguez, Arun Kumar Joshi, Hans Joachim Braun, Ravi Prakash Singh

**Affiliations:** ^1^Global Wheat Program, International Maize and Wheat Improvement Center (CIMMYT), Texcoco, Mexico; ^2^Colegio de Post-Graduados, Texcoco, Mexico; ^3^Instituto Nacional de Investigaciones Forestales, Agricolas y Pecuarias (INIFAP), Campo Experimental Valle de México, México, Mexico; ^4^Global Wheat Program, International Maize and Wheat Improvement Center (CIMMYT), New Delhi, India; ^5^Programa de Protección Vegetal, Universidad Autónoma Chapingo, Texcoco, Mexico

**Keywords:** genotype-by-environment interaction, response to selection, genetic correlations, multi- environmental trials, pedigree-based predictions

## Abstract

In this study, we defined the target population of environments (TPE) for wheat breeding in India, the largest wheat producer in South Asia, and estimated the correlated response to the selection and prediction ability of five selection environments (SEs) in Mexico. We also estimated grain yield (GY) gains in each TPE. Our analysis used meteorological, soil, and GY data from the international Elite Spring Wheat Yield Trials (ESWYT) distributed by the International Maize and Wheat Improvement Center (CIMMYT) from 2001 to 2016. We identified three TPEs: TPE 1, the optimally irrigated Northwestern Plain Zone; TPE 2, the optimally irrigated, heat-stressed North Eastern Plains Zone; and TPE 3, the drought-stressed Central-Peninsular Zone. The correlated response to selection ranged from 0.4 to 0.9 within each TPE. The highest prediction accuracies for GY per TPE were derived using models that included genotype-by-environment interaction and/or meteorological information and their interaction with the lines. The highest prediction accuracies for TPEs 1, 2, and 3 were 0.37, 0.46, and 0.51, respectively, and the respective GY gains were 118, 46, and 123 kg/ha/year. These results can help fine-tune the breeding of elite wheat germplasm with stable yields to reduce farmers’ risk from year-to-year environmental variation in India’s wheat lands, which cover 30 million ha, account for 100 million tons of grain or more each year, and provide food and livelihoods for hundreds of millions of farmers and consumers in South Asia.

## Introduction

Among the world’s three most important staple food crops, wheat is grown on more than 215 million hectares (ha) worldwide, producing over 735 million tons (t) of grain (USDA, 2020). In South Asia, wheat is consumed by more than 1.8 billion people and is grown on about 47 million ha with an annual production of almost 145 million t (USDA, 2020). More specifically, India is the region’s largest wheat producer, with 30 million ha of wheat area accounting for 107 million t of grain (USDA, 2020).

The International Maize and Wheat Improvement Center (CIMMYT) annually develops and distributes improved wheat germplasm to hundreds of partners worldwide, free of charge in the form of targeted yield trials and observation nurseries. Recipients grow the trials and nurseries, keep promising lines for their breeding programs or for direct release and are asked to return performance data that are collectively interpreted and shared to guide further breeding. The data reflect significant variations in growing environments across years and locations and serve as an input for CIMMYT wheat breeding, resulting in lines that are diverse and specially selected for yield potential, disease resistance, end-use quality, and climate resilience. Research has shown that conditions at the CIMMYT wheat research station near Ciudad Obregón, an irrigated desert farm in northwestern Mexico, correlate with diverse wheat-growing environments worldwide (DeLacy et al., 1993; Lillemo et al., 2005) and, by controlling irrigation, create a simulation of six selection environments (SEs) that represent major CIMMYT target regions for wheat breeding and deployment in the developing world.

As part of CIMMYT’s efforts to describe, measure, and analyze genotype × environment (GE) interaction in the multi-environment testing of wheat lines, Cooper and DeLacy (1994) identified three areas: analysis of variance, indirect selection, and pattern analysis. Pattern analyses in the form of classification and ordination methods (Cooper and Hammer, 1996) involve developing biplots for the first two or three principal components for lines and environments to portray the relationships among environments, whereby the representation of discrimination among lines from the classification can be superimposed on the low-dimensional space defined by the ordination. Statistical applications by CIMMYT and partners to assess GE in extensive international wheat trials found that CIMMYT’s main testing location can be associated with various international sites (Crossa, 1990; Crossa et al., 1991; Cooper et al., 1993a, b, c, d) within certain mega-environments (MEs) that represent the world’s major wheat-producing areas (Rajaram et al., 1994; Braun et al., 1996).

A target population of environments (TPE) is a variable group of future production environments. A TPE is composed of many environments and future years or growing seasons. Naturally, GE may result from relatively static and predictable variation—for example, in soil or other conditions across the field—and dynamic, unpredictable, and often significant temporal variability—i.e., weather over different years.

The TPE delineations are based on climate, soil, and hydrological characteristics and can also include socioeconomic features, such as the resource levels of farm households. There are also different ways to group trials and environments into a TPE. Data from environmental sensors and satellites can be used to develop stratified hierarchical cluster analyses (Cooper and Hammer, 1996) of sites and thus identify homogeneous environments wherein line performances will be highly correlated.

SEs are where a breeder selects lines. The SE is identified as predicting the performance in a given TPE, but the SE may not itself belong to the TPE. To determine if lines tested in the SE are useful to predict the performance of those in the TPE, it is important to (1) compute the genetic correlations between the lines in the SE vs. the same (or related) lines in the TPE and show relatively high correlations between performances in both; (2) screen lines in the SE, with the repeatability (broad-sense heritability), where the SE is higher than the TPE; and (3) ensure that the SE allows a large number of lines to be screened at a low cost, such that the SE provides a high selection intensity.

In this study, we aimed to define TPEs for wheat breeding in India using historical meteorological and soil data and to estimate the genetic correlation, the direct and correlated response to selection between the TPEs and the SE of Ciudad Obregón, the prediction ability of the SE in Mexico, and the grain yield (GY) gains in each TPE over time.

## Materials and Methods

### Data

We used GY data from crop cycles 2001–2002 to 2016–2017 of Elite Spring Wheat Yield Trials (ESWYT) nurseries tested in India and at the CIMMYT-Ciudad Obregón research station (27° 37’ N, altitude 39 masl, average annual rainfall 330 mm). Trials were conducted under five SEs:

(1) Beds with five irrigations (B5IR): Trials were grown on raised beds with full (optimal) irrigation, meaning about 500 mm of available water and an optimal sowing date of late November–mid December.
(2) Flat five irrigations (F5IR): Trials grown on the flat with optimal irrigation and optimal sowing date.
(3) Beds with two irrigations (B2IR). Trials were grown on raised beds with partial irrigation of about 260 mm of available water and optimal sowing date.
(4) Flat drought (FDRT): Planted trials were grown on the flat with about 180 mm of available water, creating severe drought conditions, and optimal sowing date.
(5) Beds late heat (BLHT): Trials were sown in February, a non-optimal planting month that subjects the crop to terminal heat stress, and optimally irrigated.

The 41 trial locations across India (Table 1) provided 245 site–year combinations. The ESWYT is distributed yearly on request and consists of 49 genotypes plus a local check included by cooperators at the trial site. The genotypes included are selected after 2 or 3 years of testing at CIMMYT-Ciudad Obregón. CIMMYT generates some 9,000 new wheat lines each year that are tested between November and April under optimal conditions (Stage 1) at the Ciudad Obregón station. From those, 1,000 selected lines are tested in six simulated environments (Stage 2) that include varying levels of drought and high-temperature stress, as well as optimal conditions. About 250 lines from Stage 2 are advanced for further testing in three of the six simulated environments (Stage 3). At that point, seed from the lines is also multiplied in a Karnal bunt-free area in Mexico for use in ESWYT and other trials and nurseries. ESWYT entries change each year, resulting in a lack of connectivity between years of evaluation. Data are not provided for 2002–2004 because the trials were not grown in the described SEs in those years.

**TABLE 1:** Locations in India considered for the definition of Target Population of Environments (TPE) with Elite Spring Wheat Yield Trials (ESWYT) germplasm.

Description	Institute name	Latitude	Longitude	TPE
Chandpa_GKSPL	Ganga Kaveri Seeds Pvt Ltd.	27.58	78.05	1
DWR_dplantbreedingg	DWR	32.1	76.05	1
DWR_Karnal1	SKUAST-J	29.67	77.03	1
DWR_Karnal2	DWR	29.67	76.97	1
Hissar	CCS Haryana Agricultural Univ.	29.17	75.77	1
Jalander_SB	Bioseed Research India Ltd.	30.90	75.8	1
Ludh_BISA	BISA	30.95	75.88	1
Mahyco	MAHYCO	29.95	76.88	1
Naruana_MHS	MAHYCO	30.2	74.93	1
Newdelhi_IARI	IARI-Div. Of Genetics	28.58	77.2	1
NSL_Guargaon	Nuziveedu Seeds Ltd.	28.62	77.07	1
Pantnagar	G.B. Pant Univ. Of Agr.	29	79.5	1
PAU_Gurdaspur	Punjab Agricultural University	32.03	75.4	1
PAU_Ludh	Bioseed Research India Ltd.	30.93	75.87	1
Syngenta_Karnal	Syngenta India Limited	29.63	75.1	1
Azadu_ATK	C.S.S. Research Institute	26.47	80.4	2
BAU_Ranchi	DWR	23.35	85.32	2
Bhagalpur	Bihar Agricultural University	25.23	87.07	2
BHU	Banaras Hindu University	25.3	83.05	2
Ghajipur	S.D. Agricultural University	26.78	82.2	2
IARI_PUSA	Univ. Of Ag. & Tech.	25.87	85.8	2
IRRS_Bilaspur	Birsa Agricultural University	22.15	82.2	2
Pusa_BISA	BISA	25.88	85.82	2
AFH_Pune	Agharkar Res. Institute	18.07	74.35	3
Ankurrfk_Nagpur	Ankur Seeds Pvt. Ltd.	21.15	79.15	3
ARI_Jaipur	Agharkar Res. Institute	26.97	75.8	3
Dbotany	Deemed University	27.98	78.97	3
GAU_Junagadh	Junagarh Agricultural Univ.	21.5	70.48	3
GAU_Vijapur	S.D. Agricultural University	23.58	72.75	3
Gokulwadi_Jalna	Krishidhan Seeds Private Ltd.	19.85	75.88	3
Gwalior	DWR	26.22	78.23	3
Hoshangabad	Junagarh Agricultural Univ.	22.73	77.7	3
Indore	IARI	22.62	75.83	3
ITC_Vidisha	ITC Life Scs & Technology Ctr	23.52	77.82	3
Jab_BISA	BISA	23.17	79.98	3
Jabalpur_LFJ	JNKVV	23.15	79.97	3
KSPL_Ganhinagar	Krishidhan Seeds Pvt. Ltd.	23.25	72.75	3
MPKV_Niphad	Mahatma Phule Krishi Vidyapeet	20.1	74.1	3
RDW_ugarsugar	Univ. Of Agr. Scs.	15.73	75.18	3
UAS_Dharwad	Univ. Of Agric. Sciences	15.7	76.12	3
UGAR_SUGAR_FRF	Univ. Of Agric. Sciences	15.43	75.12	3

### Definition of the Target Population of Environments

Daily data from 2003 to 2016 for nine meteorological variables (MVs) for locations in India (Table 1) were obtained from the NASA Langley Research Center (LaRC) POWER Project funded through the NASA Earth Science/Applied Science Program. In addition, nine soil-related variables (SVs) for each location were obtained through the Soil Grids application of the International Soil Reference and Information Centre. The MVs and SVs are listed in Table 2. Each MV was averaged over periods of 20 days starting from November 23 each year and reaching 120 days. The starting date used was the median in the range of planting dates in India.

**TABLE 2:** Meteorological variables (MVs) and soil-related variables (SVs) considered for Target Population of Environments (TPE) definition in India.

			TPE		
Type	Variable	Units	1	2	3
MV	Average temperature at two meters	°C	15.82	18.7	21.43
MV	Maximum temperature at two meters	°C	24.26	27.27	30.69
MV	Minimum temperature at two meters	°C	9.5	11.69	13.72
MV	Cooling degree days	°C-d above 0 °C	16.88	19.48	22.2
MV	Dew point	°C	0.65	6.18	4.24
MV	Precipitation	mm	12.76	7.83	4.37
MV	Relative humidity at two meters	In %	38.29	46.87	35.77
MV	Clear sky insolation incident on a horizontal surface	kW-h/m^2^/day	−18.47	−13.12	7.68
MV	Wind speed at two meters	m/s	1.59	1.68	1.86
SV	Available soil water capacity	Volumetric fraction in %	15.2	13.12	8.72
SV	Available soil water capacity until wilting point	Volumetric fraction in %	20.4	22.87	27.5
SV	Cation exchange capacity of soil	cmolc/kg	14.47	18.75	35.5
SV	Coarse fragments volumetric	In %	5.467	7.75	15.4
SV	Soil organic carbon density	kg/m^3^	94.27	118.37	124
SV	Soil pH in H_2_O	pH	7.53	6.91	7.43
SV	Clay content (0–2 μm)	Mass fraction in %	25.2	30	40.4
SV	Sand content (50–2,000 μm)	Mass fraction in %	37.4	32.2	32.3
SV	Silt content (2–50 μm)	Mass fraction in %	37.4	38	27.5

For each location, 63 MV and SV variables were used for principal component analysis (PCA) with the correlation matrix of the data to infer the number of groups (TPEs) that explain most of the variation. We then conducted hierarchical clustering of the groups based on Euclidean distance, according to Ward (1963). The cluster solution (k) was equal to the number of groups identified in the PCA.

The TPEs were plotted on a map of India and interpolated by fitting a Thin Plate Spline regression (Wood, 2003), with a model to fit:

(1) $$y_{i}=f\left(x_{i}\right)+\varepsilon_{i}$$

where *y_i_* is the response variable, *x_i_* are covariates, *f*() is a *d* dimensional surface smoothing function, and $\varepsilon_{i}^{\prime }s$ are model residuals distributed as independent and identically distributed random variables with mean 0 and variance σ^2^. Thin plate splines were used to estimate *f* by finding the function *g* that minimizes:

(2) $${∥\text{y}-\text{g}∥}^{2}+\lambda J_{md}\left(g\right)$$

where **y** is the vector of data, **g** = (*g*(*x*_1_),*g*(*x*_2_)*g*(*x*_3_),…,*g*(*x*_*n*_))′, *J*_*md*_(*g*) is a penalty functional that measures the change of *g*, and λ is a smoothing parameter.

### Trial Analysis in India Target Population of Environments

After defining India TPEs using environmental data, we performed a multi-environment trial analysis of GY by year and TPE. The following model was used to estimate variance components:

(3) $$\text{y}=\mu +{\text{E}}_{s}+{\text{R}}_{j}+{\text{R}}_{j}\left({\text{E}}_{s}\right)+{\text{G}}_{i}+\text{G}E_{ij}+\varepsilon_{ijs}$$

where μ is the general mean, **E_s_** is the fixed effect of the environments (s = 1, …, n), **R_j_** is the random effects of the replicates (*j* = 1, 2), **G_i_** is the random effects of the genotypes (*i* = 1,…, 50), **G***E*_**i***j*_ represents the random effect of GE interaction, and ε_*ijk*_ is a random residual assumed to be independently, identically, and normally distributed (IID) with mean zero and variance $\sigma {}_{e}^{2}$.

Additionally, we fitted another mixed model to include the TPEs as a fixed effect with:

(4) $$\begin{array}{c} \text{y}=\mu +\text{T}PE_{k}+{\text{G}}_{i}+\text{G}*\text{T}PE_{ik}+\text{G}*{\text{E}}_{is}\left(\text{T}PE_{k}\right)+{\text{E}}_{s}\left(\text{T}PE_{k}\right)+\\ {\text{R}}_{j}\left({\text{E}}_{s}\right)+\text{S}B_{l}\left(\text{E}*{\text{R}}_{sj}\right)+\varepsilon_{ijkls} \end{array}$$

where **T***PE*_**k**_ is the fixed effect of the TPEs (k = 1, 2, 3), and **S***B*_**l**_ represents the random effects of sub-blocks in the alpha-lattice design (l = 1, …, 5), and the remaining terms are as in Equation (3).

The broad-sense heritability (*H*^2^) of the trials across environments in each TPE–year combination and across environments, and TPE was calculated with Equations (5) and (6), respectively:

(5) $$H^{2}=\frac{V_{g}}{V_{g}+\frac{V_{ge}}{ne}+\frac{V_{r}}{nr\times ne}}$$

(6) $$H^{2}=\frac{V_{g}+V_{g*tpe}}{V_{g}+V_{g*tpe}+\frac{V_{ge\left(tpe\right)}}{ne}\frac{V_{r}}{nr\times ne}}$$

where *V_g_* represents the genotypic variance, *V*_*ge*_ represents the GE variance, *V_r_* is the residual variance, and *ne* and *nr* are the number of environments and replicates, respectively. In Equation (6), *V*_tpe*g_ represents genotype × TPE variance, *V*_*ge(tpe)*_ is the variance of the GE within TPEs.

### Trial Analyses in Selection Environments of Mexico

The trials conducted in the SEs were analyzed individually with the following model:

(7) $$\text{y}=\mu +{\text{R}}_{j}+{\text{SB}}_{k}\left({\text{R}}_{j}\right)+{\text{G}}_{i}+\varepsilon_{ijk}$$

where *μ* is the general mean, **G_i_** is the random effects of the genotypes (*i* = 1, …, 50), **R_j_** is the random effects of the replicates (*j* = 1, 2), **SB_k_** denotes the random effects of the sub-blocks (*k* = 1, …,5) assumed to be independently, identically, and normally distributed (IID) with mean zero and variance *σ^2^_*s*__*b(r)*_*, and ε_*ijk*_ is a random residual assumed to be IID with mean zero and variance $\sigma_{e}^{2}$. The results from this model were used to conduct pedigree-based predictions.

The mixed model fit for the across-SE analysis was:

(8) $$\text{y}=\mu +\text{S}E_{s}+{\text{R}}_{j}+\text{R}*\text{S}B_{jk}\left(\text{S}E_{s}\right)+{\text{G}}_{i}+\text{G}*\text{S}E_{is}+\varepsilon_{ijks}$$

where **S***E*_**j**_ represents the SEs (s = 1, …, 5), and the remaining terms are as in Equation (7).

The heritability of trials in SE in Equation (8) was estimated with Equation (5), but replacing *V*_*ge*_ with the genotype × SE interaction and *ne* with the number of SE.

### Correlation Between Target Population of Environments and Selection Environment

Once we determined variance components by year, across sites in TPEs, and across SE and averaged them across years, the same models were fitted using the genotypes and the GE for TPEs as fixed effects to make best linear unbiased estimations (BLUEs) and subsequently calculate Pearson’s correlation coefficient between each TPE–year combination and across SE–year combinations. The respective estimation of genetic correlations was then computed according to Cooper and DeLacy (1994):

(9) $$r_{ij}=\frac{p_{ij}}{\sqrt{h_{i}^{2}\times h_{j}^{2}}}$$

where *r*_*ij*_ is the genetic correlation, *p*_*ij*_ represents the phenotypic correlation between environments or groups of environments *i* and *j*, and $h_{i}^{2}$ and $h_{j}^{2}$ are the heritability of environments or groups of environments *i* and *j*, respectively.

### Estimation of the Correlated and Direct Response to Selection

Once the genetic correlation by year was estimated, and assuming the same selection intensity in TPE and SE, the correlated response to selection (*CR*) was then calculated following Falconer (1952) and Searle (1965):

(10) $$CR=\bar{r}\times \sqrt{\frac{H_{SE}^{2}}{H_{TPE}^{2}}}$$

where $\bar{r}$ is the average genetic correlation (Equation 9) of years of testing between TPEs and across SEs, $H_{SE}^{2}$ is the broad-sense heritability across the SE, and $H_{TPE}^{2}$ is the broad-sense heritability across sites of the TPE. Both heritability terms in Equation (7) were averaged over years of testing in TPEs and SEs. The correlated response (CR) measures the relative efficiency of selecting indirectly for the TPEs by using the information across SE.

The expected direct response to selection (*DR*) across sites without considering TPEs, across SEs, and across sites in each TPE was estimated with Equation (11), dividing the genetic variance by the square root of phenotypic variance, disregarding selection intensity (*i* = 1).

(11) $$DR=i\frac{V_{g}}{\sqrt{V_{g}+\frac{V_{ge}}{ne}}+\frac{V_{r}}{ne\times nr}}$$

where the variance terms represent the same as those in Equation (5). The DR measures the estimated response in the TPE or across SEs by directly selecting in those same environments.

### Pedigree-Based Predictions of India Target Population of Environments Using the Mexico Selection Environment

We estimated the pedigree-based prediction accuracy of the Mexico SE for the 2016 cycle of the ESWYT in each TPE. The training population consisted of the lines in Stage 1 that were selected for Stages 2 and 3 plus their subsequent evaluations in the last two stages of yield testing in the SE. We also incorporated the ESWYT data from each SE and TPE over 2010–2015. The preceding gave a total of 3,599 unique lines and 22,872 line–year–environment combinations.

We used three models to predict the performance of the wheat lines at Indian sites of the TPE using Mexico’s SE.

#### Model 1 (AE)

This model incorporates random effects of the pedigree information along with environments:

(12) $$y_{ij}=\mu +E_{i}+a_{j}++\varepsilon_{ij}$$

where *E_i_* is the random effect of environment (TPE/SE–year combination), assumed to be IID with mean zero and variance $\sigma_{E}^{2}$; *a_j_* is the random effect of lines with the pedigree information we assume **a**∼*MN*(**0**, $\sigma_{a}^{2}\text{A})$, where $\sigma_{a}^{2}$ is the additive variance parameter, *A* is the additive relationship matrix derived from pedigree, and *MN* stands for multivariate normal. The additive relationship matrix was calculated with the R package pedigree (Coster, 2012).

#### Model 2 (AE-AxE)

We added the random effect of the interaction between lines and environments to Model 1:

(13) $$y_{ij}=\mu +E_{i}+a_{j}+aE_{ij}++\varepsilon_{ij},$$

where *AE*_*ij*_ is the random effect of the interaction between lines and environments, assuming that the joint distribution of interaction vector $\text{a}E∼MN({\left(0,{\text{Z}}_{p}{\text{AZ}}_{p}^{{}^{\prime }}\right)}^{\circ }\left({\text{Z}}_{E}{\text{Z}}_{E}^{{}^{\prime }}\right)\sigma_{aE}^{2}$), where **Z**_*p*_ is the matrix linking the phenotypic entries with the pedigree information, **Z**_*E*_ is the incidence matrix of environmental effects, and ° represents the Hadamard product between matrices to impose the reaction norm structure on GE (Jarquín et al., 2014; Pérez-Rodríguez et al., 2015).

#### Model 3 (AE-AxE-W-AxW)

Model 3 is an extension of Model 2 that incorporates an additional random effect of MVs and their interactions with the lines of each TPE.

(14) $$y_{ij}=\mu +E_{i}+a_{j}+aE_{ij}+t_{ij}+at_{ij}+\varepsilon_{ij},$$

where tij=∑qWγqijq with **W** being a matrix of environmental covariates and γ_*q*_ representing the effect of the environmental covariates, which we assume as independent and identically distributed random variables with mean zero and variance $\sigma_{\gamma }^{2}$, and we further assume that $\text{a}t∼MN\left(0,{\left({\text{Z}}_{p}{\text{AZ}}_{p}^{{}^{\prime }}\right)}^{\circ }\left({\text{WW}}^{\prime }\right)\sigma_{aw}^{2}\right)$ (Jarquín et al., 2014; Pérez-Rodríguez et al., 2015).

In our implementation, phenotypic data and MVs were centered and scaled to unit variance. The models were run in the BGLR (Pérez and de los Campos, 2014) package for R (R Development Core Team, 2020). The Pearson’s correlation coefficient between the predicted and observed values in the TPE were used as an indicator of the predictive ability of the SEs for each model.

### Grain Yield Gain Estimations in Target Population of Environments

We used the BLUEs from Equation (3) to fit the reaction norm model from Equation (12) with the additive relationship matrix and then regressed the best linear unbiased predictions (BLUPs) from Equation (12) for each TPE in India on the years when ESWYTs were grown. The slope of the regression was taken as the gain in GY in TPEs.

## Results

### Definition of Target Population of Environments

**FIGURE 1:**
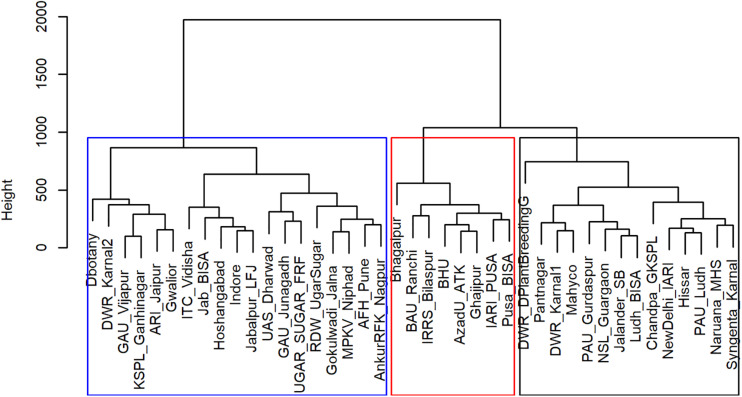
Hierarchical clustering of Elite Spring Wheat Yield Trials sites in India based on meteorological and soil variables.

**FIGURE 2:**
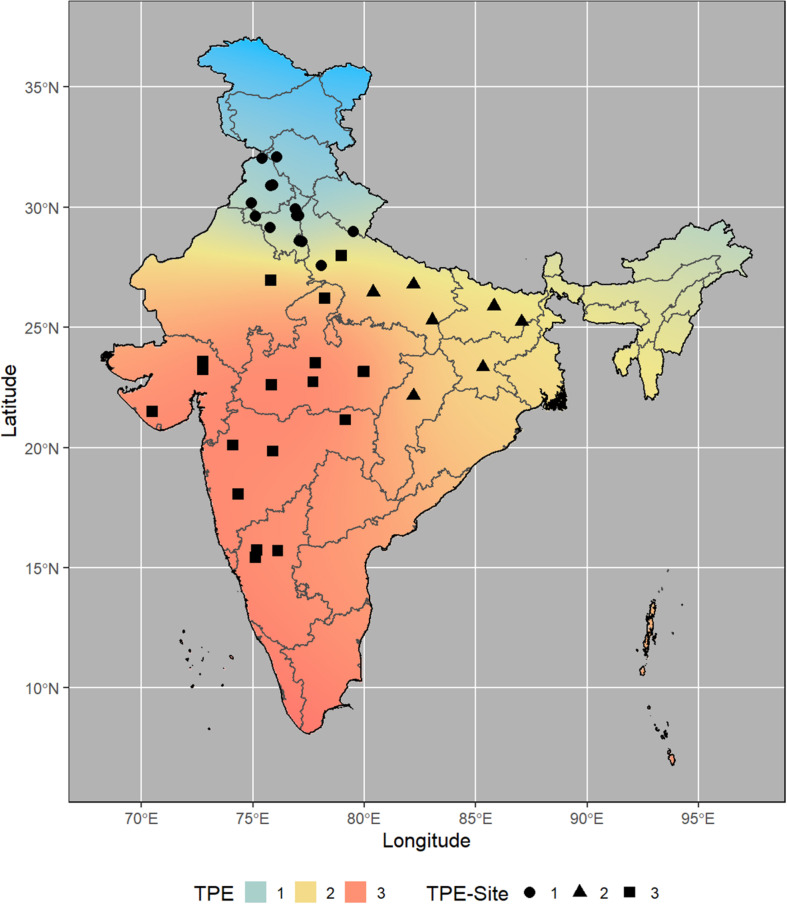
Location of Elite Spring Wheat Yield Trials sites within each Target Population of Environments (TPE) in India.

From the analysis of MVs and SVs, we obtained three main clusters (TPEs) of locations (Figure 1) that explained most of the variations (>70%) in the data set (Table 1 and Figure 2). These TPEs tended to be concentrated in three main geographical zones of India: TPE 1, representing the Northwestern Plain Zone (NWPZ); TPE 2, the North Eastern Plains Zone (NEPZ); and TPE 3, the Central-Peninsular Zone (CPZ) (Figure 2). The respective proportion of sites in each TPE above was 36.6, 19.5, and 43.9%.

Average temperatures were lowest in TPE 1 (about 16°C) and highest in TPE 3 (about 21°C), with TPE 2 in between the two (about 19°C) (Table 2). Cooling degree days and dew point followed a similar pattern (TPE 1 < TPE 2 < TPE 3).

The average accumulated precipitation during the period was roughly 12.8, 7.8, and 4.4 mm for TPEs 1, 2, and 3, respectively (Table 2). Average relative humidity was highest in TPE 2 (47%) and recorded at 38 and 36% in TPEs 1 and 3, respectively. Clear sky insolation incidence (kW-h/m^2^/day) was −18.47, −13.12, and 7.68, for TPEs 1, 2, and 3, respectively. Average wind speed at 2 m height did not exceed 2 m/s in the three TPEs.

Regarding soil conditions (Table 2), the average available soil water capacity was highest in TPE 1 (15%) and lowest in TPE 3 (9%), whereas available soil water capacity at wilting point was highest in TPE 3 (28%) and lowest in TPE 1 (20%). The average cation exchange capacity was at its highest in TPE 3 (35.5 cmolc/kg)—roughly double the values in the other TPEs—a similar pattern was observed for coarse fragments (TPE 3: 15%); TPE 3 also had the greatest soil organic carbon density (124%), although the values for TPEs 2 and 1 followed closely. Soil water pH ranged from 7.75 (TPE 1) to 6.92 (TPE 2). Soil texture compositions (clay-sand-silt) were fairly balanced and differed slightly for TPEs, with TPE 3 having more clayey soils.

### Variance Components, Genetic Correlations, and Correlated and Direct Response to Selection

Average GYs across years and sites ranged from 3.7 t/ha (TPE 2) to 5.1 t/ha (TPE 1). The average GY across sites for India was 4.7 t/ha, whereas the average GYs across years and SE were 7.1, 4.8, 3.7, 6.7, and 2.5 t/ha for B5IR, B2IR, BLHT, F5IR, and FDRT, respectively.

The average of the variance components indicates that the size of the G^∗^TPE variance relative to that of the G^∗^E(TPE) was 7.3%. The size of the variance of G^∗^E(TPE) relative to the genotypes (G) was 518% (Table 3). The size of the G^∗^TPE variance was 38% of the G variance. The *H*^2^ estimated from the average of the variance components was 0.72, and the average *DR* estimated from the variance components was 0.18 (Table 3).

**TABLE 3:** Variance components, phenotypic variance (V_*Z*_), heritability (H^2^), genetic correlation (r), correlated response (CR), and direct response to selection (DR) of Elite Spring Wheat Yield Trials (ESWYT) grown from 2001 to 2016 across sites and Target Population of Environments (TPEs).

Year	ESWYT	E(TPE)	G	G*TPE	G*E(TPE)	R(E)	SB(E*R)	Residual	V_*Z*_	H^2^	r	CR	DR
2001	22	4.0036	0.0783	0	0.1008	0.01097	0.0731	0.2612	0.10	0.77			0.25
2005	26	0.7807	0.0312	0.01168	0.1298	0.00358	0.05208	0.2705	0.07	0.62	0.42	0.41	0.16
2006	27	1.5786	0.0318	0	0.1981	0.0332	0.09257	0.201	0.05	0.60	0.55	0.49	0.14
2007	28	1.0941	0.0222	0.03947	0.1228	0.01797	0.03856	0.1703	0.08	0.79	0.47	0.50	0.22
2008	29	3.3262	0.0126	0	0.1749	0.02885	0.07857	0.09306	0.03	0.41	0.41	0.36	0.07
2009	30	0.5347	0.0169	0.00117	0.1189	0.00566	0.02894	0.108	0.03	0.59	0.54	0.49	0.10
2010	31	0.731	0.0303	0.01759	0.1847	0.00575	0.02445	0.2474	0.07	0.73			0.19
2011	32	2.4407	0.0443	0.00755	0.1826	0	0.07045	0.2638	0.06	0.80	0.70	0.57	0.20
2012	33	1.1673	0.0392	0.01684	0.2073	0.00638	0.03984	0.1878	0.07	0.80	0.51	0.47	0.21
2013	34	2.1213	0.0163	0.02693	0.28	0.05409	0.04214	0.2104	0.07	0.66	0.90	0.89	0.17
2014	35	0.6268	0.0344	0.0083	0.1122	0.00852	0.04085	0.1364	0.05	0.79	0.54	0.48	0.18
2015	36	0.632	0.0242	0.01403	0.1412	0.00112	0.03473	0.1841	0.06	0.68	0.28	0.24	0.16
2016	37	2.2357	0.022	0.00963	0.1416	0.02188	0.03929	0.2225	0.04	0.70	0.56	0.52	0.15
**Average**		1.6364	0.0311	0.0118	0.1611	0.0152	0.0504	0.1967	0.06	0.72	0.53	0.50	0.17

*E, Variance of Environments (Sites); G, Genotypic variance; R, Reps; SB, Sub-blocks.*

On the other hand, the relative size of the variance of G in each TPE to that of the across-site analysis was 1.8, 2.4, and 1.1 times higher in TPEs 1, 2, and 3, respectively (Tables 3, 4). The size of the G^∗^E variance in each TPE relative to that of G in its respective TPE was 290, 184, and 421% times higher. The average phenotypic and genetic correlation between TPEs was low and medium size, respectively, ranging from 0.26 to 0.31 (phenotypic) and 0.56 to 0.63 (genetic) (Table 5).

**TABLE 4:** Average variance components of Elite Spring Wheat Yield Trials (ESWYT) grown from 2001 to 2016 by Target Population of Environments (TPE).

TPE	G	E	G*E	R*SB(E)	SB(R)	Residual	Average sites
1	0.056	1.342	0.164	1.405		0.232	6.8
2	0.075	1.074	0.138	1.611	0.094	0.167	2.5
3	0.033	1.855	0.139	1.954		0.202	5.1

*G, Genotypic variance; E, Variance of Environments (Sites); R, Reps; SB, Sub-blocks.*

**TABLE 5:** Average phenotypic and genetic correlations between Target Population of Environments (TPEs).

Phenotypic correlation				
	1	2	3	
1	1	0.29	0.31	
2		1	0.26	
3			1	

**Genetic correlation**				

	**1**	**2**		**3**

1	1	0.56		0.63
2		1		0.60
3				1

Regarding variance components across SEs (Table 6), the variance of G is 2.3 times higher than that of G^∗^SE (Table 6). Also, G is 3.3 times greater than the one across sites of the TPEs, and its size relative to each TPE was 1.8, 1.4, and 3.1 for TPEs 1, 2, and 3, respectively (Tables 4, 6). The *H*^2^ estimated from the average variance components was 0.73, which is about the same as the one across all sites in the TPEs, and the magnitude of *H*^2^ in the SEs relative to TPEs 1, 2, and 3 was, respectively 1.2, 2.1, and 2.1 times higher. The estimated DR across SE was 0.28, which is 1.6 times higher than that of the DR across TPEs, whereas it was 1.6, 1.7, and 2.5 times higher than that of the TPEs 1, 2, and 3, respectively (Tables 6, 7).

**TABLE 6:** Variance components, phenotypic variance (V_*Z*_), heritability (H^2^) across selection environments (SEs), and direct response to selection (DR) of Elite Spring Wheat Yield Trials (ESWYT) grown from 2001 to 2016 across SEs.

Year	ESWYT	G	G*SE	R*SB	R*SB(SE)	Residual	Var_*Z*_	H^2^	DR
2001	22	0.4496		0.15857		0.40665	0.65		0.56
2005	26	0.18293	0		0.09811	0.37411	0.28	0.66	0.35
2006	27	0.17917	0.06219		0.36282	0.2721	0.28	0.64	0.34
2007	28	0.05154	0.0706		0.14638	0.10379	0.08	0.63	0.18
2008	29	0.02129	0.05575		0.12668	0.07947	0.04	0.53	0.11
2009	30	0.03146	0		0.05682	0.10463	0.05	0.64	0.14
2010	31	0.1926		0.00927		0.18273	0.28		0.36
2011	32	0.03193	0.02453		0.06503	0.08938	0.06	0.58	0.14
2012	33	0.09795	0.06943		0.09896	0.04184	0.14	0.68	0.26
2013	34	0.02496	0.06268		0.08571	0.08912	0.05	0.48	0.11
2014	35	0.03325	0.02416		0.08932	0.07997	0.05	0.67	0.15
2015	36	0.02962	0.07571		0.08995	0.11402	0.06	0.53	0.12
2016	37	0.03387	0.04699		0.06042	0.05311	0.05	0.65	0.15
Mean		0.1046	0.0447	0.0839	0.1164	0.1531	0.14	0.73	0.28

*G, Genotypic variance; E, Variance of Environments (Sites); R, Reps; SB, Sub-blocks.*

**TABLE 7:** Phenotypic variance (V_*z*_), heritability for the TPE (H^2^_*t*__*pe*_), genetic correlations (r), correlated response (CR), by selecting across selection environments and direct response (DR) to selection in each Target Population of Environments (TPE).

		TPE 1					TPE 2					TPE 3				
Year	ESWYT	V_*z*_	H^2^_*t*__*pe*_	r	CR	DR	V_*z*_	H^2^_*t*__*pe*_	r	CR	DR	V_*z*_	H^2^_*t*__*pe*_	r	CR	DR
2001	22	0.14	0.3			0.11	0.15	0.67			0.26	0.09	0.34			0.1
2005	26	0.11	0.54	0.65	0.72	0.18	0.06	0.64	0.57	0.58	0.16	0.10	0.2	–0.09	–0.16	0.06
2006	27	0.07	0.41	0.38	0.48	0.11	0.2	0.22	0.99	1.70	0.1	0.05	0			0.00
2007	28	0.06	0.57	0.39	0.41	0.14	0.72	0.44	0.84	1.01	0.37	0.10	0.58	–0.04	–0.04	0.18
2008	29	0.05	0.22	0.9	1.55	0.05	0.16	0.2	0.06	0.10	0.08	0.05	0			0.00
2009	30	0.04	0.46	0.42	0.50	0.1	0.16	0.2	0.99	1.79	0.08	0.06	0.33	0.14	0.20	0.08
2010	31	0.14	0.56			0.21	0.08	0.25			0.07	0.08	0.46			0.13
2011	32	0.12	0.69	0.99	0.83	0.24	0.08	0.14	0.45	0.92	0.04	0.09	0.63	0.63	0.61	0.19
2012	33	0.10	0.66	0.45	0.45	0.21	0.09	0.38	0.16	0.21	0.11	0.11	0.64	0.59	0.61	0.21
2013	34	0.14	0.64	0.82	0.71	0.24	0.12	0.06	0.99	2.86	0.02	0.08	0.18	0.7	1.13	0.05
2014	35	0.08	0.72	0.54	0.52	0.21	0.11	0.49	0.49	0.58	0.16	0.08	0.53	0.46	0.52	0.15
2015	36	0.09	0.66	0.32	0.29	0.2	0.2	0.65	0.03	0.03	0.29	0.10	0.17	0.13	0.23	0.05
2016	37	0.09	0.62	0.53	0.54	0.18	0.08	0.18	0.29	0.55	0.05	0.07	0.42	0.55	0.68	0.11
**Mean**		0.1	0.54	0.58	0.64	0.17	0.18	0.39	0.60	0.94	0.16	0.08	0.38	0.34	0.42	0.11

*ESWYT, Elite Spring Wheat Yield Trials.*

We ran the model across all sites in India, i.e., disregarding TPE subdivision, and found that the average *H*^2^ was 0.2 and the DR was 0.16. The average *H*^2^ for TPEs 1, 2, and 3 was 0.54, 0.39, and 0.38, respectively, whereas the respective *DRs* were 0.17, 0.16, and 0.11 (Table 7). The average relative selection efficiencies (CR) across SEs in each TPE were 0.6, 0.9, 0.4, and 0.5 for TPEs 1, 2, 3, and across TPEs, respectively (Tables 3, 7).

### Pedigree-Based Predictions

The proportion of additive variance relative to the total of the prediction models for each SE ranged from 1.6 to 7.9% (Table 8). The proportion of variance relative to the total (PVT) for environments varied by SE, but it remained close to 90% when the models did not include environmental covariable data or their interaction with genotypes. Inclusion of MV data reduced the PVT for the main effects of environments in all cases, which was substantial for treatments B5IR (from 92.3 to 43.2%), F5IR (from 91.7 to 41.7%), and FDRT (from 89.7 to 59.9%). In all cases, the PVTs of GEs were lower than 10% and ranged from 1.6 to 9.2% (Table 8). The PVTs of the MVs ranged from 0.2% (BLHT) to 47.8% (F5IR). The reduction in residual variance ranged from 34% (B2IR) to 56% (FDRT) in models that included the GE.

**TABLE 8:** Estimated variance components (± standard deviation of the estimator) from the pedigree-based prediction models for each selection environment (SE) in CIMMYT-Obregón and their proportion of variance relative to the total (PTV) of the models.

		Model			PTV by model (%)		
SE	Component^a^	AE	AE-AxE	AE-AxE-W-AxW	AE	AE-AxE	AE-AxE-W-AxW
B5IR	V_*A*_	0.06 ± 0.003	0.05 ± 0.003	0.05 ± 0.004	3.4	3.0	7.9
	V_*E*_	1.49 ± 0.409	1.56 ± 0.489	0.29 ± 0.127	92.4	92.1	43.2
	V_*AE*_		0.05 ± 0.003	0.04 ± 0.004		2.8	5.3
	V_*W*_			0.24 ± 0.096			36.3
	V_*AW*_			0.01 ± 0.002			1.9
	V_*r*_	0.07 ± 0.001	0.04 ± 0.002	0.04 ± 0.002	4.2	2.1	5.4
B2IR	V_*A*_	0.06 ± 0.006	0.05 ± 0.005	0.04 ± 0.006	3.8	3.2	3.0
	V_*E*_	1.37 ± 0.437	1.31 ± 0.371	1.07 ± 0.362	88.1	86.6	75.4
	V_*AE*_		0.07 ± 0.007	0.04 ± 0.006		4.7	2.7
	V_*W*_			0.15 ± 0.082			10.6
	V_*AW*_			0.03 ± 0.005			2.4
	V_*r*_	0.12 ± 0.004	0.08 ± 0.004	0.08 ± 0.004	8.0	5.4	5.9
BLHT	V_*A*_	0.07 ± 0.006	0.06 ± 0.006	0.04 ± 0.006	7.4	6.2	4.0
	V_*E*_	0.81 ± 0.241	0.76 ± 0.23	0.65 ± 0.202	80.6	77.8	74.6
	V_*AE*_		0.09 ± 0.009	0.08 ± 0.008		9.2	9.6
	V_*W*_			<0.00 ± 0.001			0.2
	V_*AW*_			0.03 ± 0.007			4.0
	V_*r*_	0.12 ± 0.003	0.07 ± 0.005	0.07 ± 0.004	12.0	6.8	7.6
F5IR	V_*A*_	0.05 ± 0.004	0.04 ± 0.004	0.03 ± 0.004	3.0	1.6	2.4
	V_*E*_	1.41 ± 0.493	2.13 ± 0.795	0.54 ± 0.324	91.7	94.1	41.8
	V_*AE*_		0.04 ± 0.004	0.02 ± 0.004		1.6	1.9
	V_*W*_			0.62 ± 0.42			47.8
	V_*AW*_			0.02 ± 0.002			1.3
	V_*r*_	0.08 ± 0.002	0.06 ± 0.003	0.06 ± 0.003	5.3	2.7	4.8
FDRT	V_*A*_	0.04 ± 0.003	0.03 ± 0.003	0.03 ± 0.004	3.3	2.1	3.6
	V_*E*_	1.07 ± 0.338	1.42 ± 0.46	0.53 ± 0.195	89.7	90.9	59.9
	V_*AE*_		0.07 ± 0.005	0.06 ± 0.005		4.6	6.6
	V_*W*_			0.22 ± 0.095			24.1
	V_*AW*_			0.01 ± 0.002			1.6
	V_*r*_	0.08 ± 0.002	0.04 ± 0.002	0.04 ± 0.002	7.0	2.3	4.2

*^a^Component: V_A_, Additive variance; V_E_, Environmental variance; V_AE_, Additive-by-Environment variance; V_W_, Environmental covariables variance; V_AW_, Additive-by-Environmental covariables variance; and V_r_, Residual Variance.*

**FIGURE 3:**
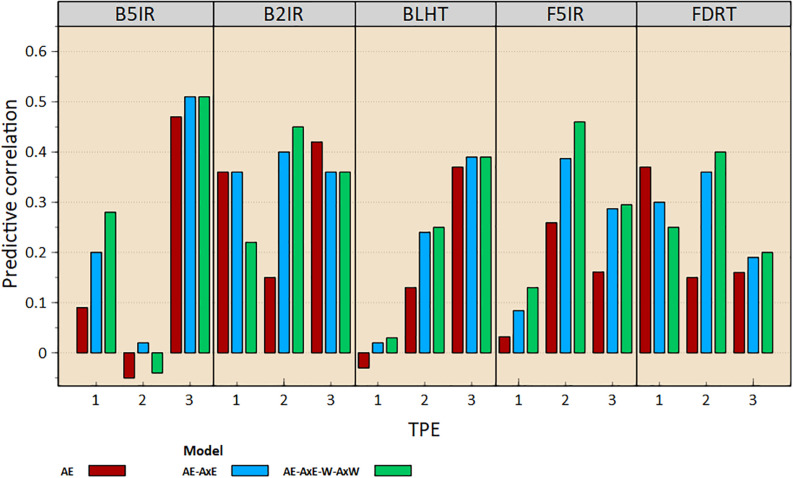
Pedigree-based predictive correlations between selection environments at the CIMMYT-Ciudad Obregón research station and a Target Population of Environments (TPE) in India for the 2016 cycle of the International Elite Spring Wheat Trial.

The predictive correlations—that is, prediction accuracies—of each SE for each TPE in the 2016 ESWYT cycle are shown in Figure 3. SEs B5IR, B2IR, and FDRT were better predictors for TPE 1. The predictive correlation of B5IR was higher for model AE-AxE-W-AxW (0.28), whereas the predictive correlation of B2IR was higher for model AE-AxE (0.36). No clear increase in predictive correlation was observed for TPE 1 when MV data were included in the model (Figure 3).

For TPE 2, in all SEs except for B5IR, the predictive correlation increased when all available information was included in the prediction model (Figure 3). The prediction accuracies between B2IR, BLHT, F5IR, and FDRT and TPE 2 were 0.4, 0.24, 0.39, and 0.36, respectively, for model AE-AxE, whereas for model AE-AxE-W-AxW, respective prediction accuracies for TPE 2 were 0.45, 0.25, 0.46, and 0.4. For TPE 3, predictive correlations with all SEs were positive (Figure 3). In all cases except B2IR, the predictive correlation increased with the inclusion of GE and/or MV data. The prediction accuracies for the AE-AxE model between B5IR, B2IR, BLHT, F5IR, and FDRT and TPE 3 were 0.51, 0.36, 0.39, 0.29, and 0.19, respectively, whereas for the AE-AxE-W-AxW, they were 0.51, 0.36, 0.39, 0.3, and 0.2, respectively.

### Grain Yield Progress in Target Population of Environments

**FIGURE 4:**
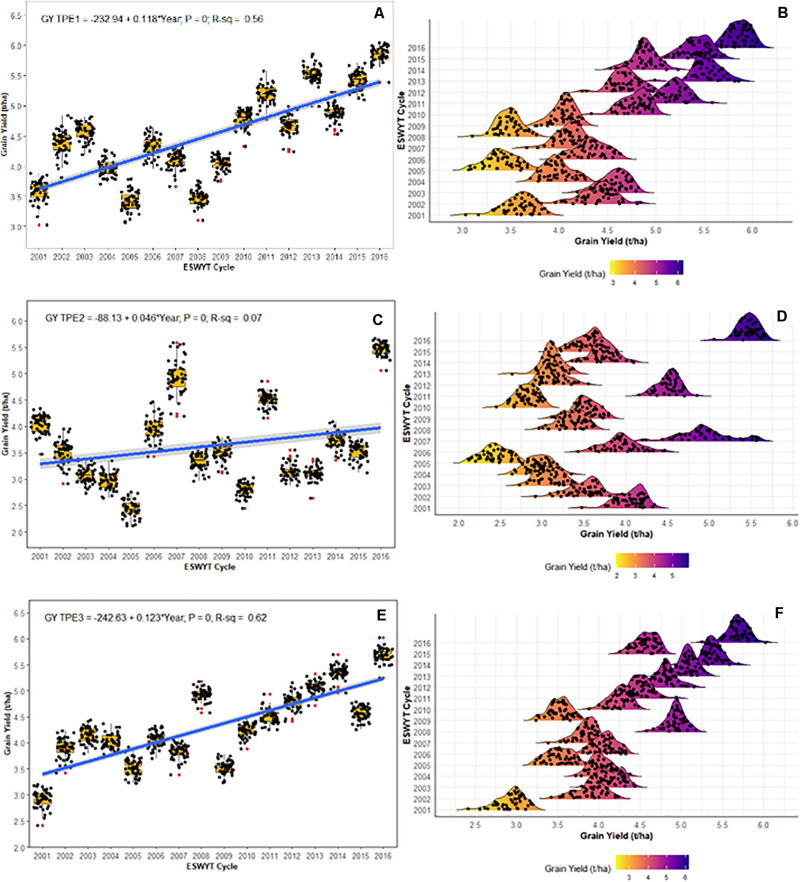
Grain yield (GY) progress and density distribution of Elite Spring Wheat Yield Trials (ESWYT) germplasm from 2001 to 2016 crop cycles in the Target Population of Environments (TPE) in India. **(A,B)** GY progress and density distribution for TPE 1; **(C,D)** GY progress and density distribution for TPE 2; **(E,F)** GY progress and density distribution for TPE 3.

The respective rates of GY gain for ESWYT germplasm in India for TPEs 1, 2, and 3 were 118 kg/ha/year (Figures 4A,B), 46 kg/ha/year (Figures 4C,D), and 123 kg/ha/year (Figures 4E,F). In all cases, the regression slope was significantly different from zero (*P* < 0.0001; Figure 4). For TPE 2, the R-squared of the regression was 0.07, providing a less clear pattern (Figures 4C,D). The density distributions (Figures 4B,D,F) depict the variation and progress of the average GY for each TPE in India, which generally tended to be lower in TPE 2 (Figure 4D) than in TPEs 1 and 3.

## Discussion

The characterization of targeted breeding regions by defining TPEs is fundamental in designing strategies for any plant breeding program (Atlin et al., 2000) and paramount for CIMMYT, which seeks to develop wheat germplasm with high and stable yields for smallholder farmers worldwide. In South Asia—home to more than 300 million undernourished people and whose inhabitants consume over 100 million tons of wheat each year—92% of the varieties released contain CIMMYT breeding contributions and half of the spring bread wheat varieties are direct releases of CIMMYT breeding lines (Lantican et al., 2016).

In the present research, we used available climate and soil data to define three main TPEs in India, the largest wheat producer in South Asia (USDA, 2020) and one of the main breeding targets for CIMMYT. These TPEs coincided with the three main geographical zones in India: TPE 1, representing the NWPZ; TPE 2, the NEPZ; and TPE 3, the CPZ. This characterization corresponds to data provided by the Government of India and represents more than 28 million ha, which is equivalent to over 97% of India’s total wheat (Singh et al., 2019).

Based on the GY data (Figure 4) and the recorded minimum temperature (Table 2), which is a critical parameter for wheat production (Asseng et al., 2015), TPE 1 is associated with optimum wheat-growing conditions, whereas wheat grown in TPE 2 and TPE 3 is subject to stress caused by higher temperatures or drought. Higher temperatures combined with higher rates of relative humidity, as occurs in TPE 2, can cause the development of leaf diseases other than rusts. Indeed, one of the most important leaf diseases in NEPZ (TPE 2) is spot blotch caused by the fungi *Bipolaris sorokiniana*, which can cause significant GY losses (Devi et al., 2018). Similarly, given the lower average temperatures in the NWPZ (TPE 1), yellow rust caused by *Puccinia striiformis* f. sp. *tritici* is currently the most relevant disease for that region, even though all three types of wheat rust occur throughout India’s wheat-growing areas (Joshi et al., 2007).

Regarding soil characteristics, TPE 1 can be classified as a loam type of soil, TPE 2 as clay loam, and TPE 3 as clay type of soil (Soil Science Division Staff, 2017). In general, the soil of the three TPEs can be considered of medium texture. The cation exchange capacity of TPE 3 indicates a higher availability of essential minerals for the plants than in TPEs 1 and 2 (Table 2). The available soil water capacity was lower in TPE 3, which may be due in part to the greater proportion of coarse fragments in its soils (Table 2), meaning that wheat crops in this environment may suffer drought stress more frequently. This can be aggravated by the suboptimal irrigation common in TPE 3 and occasionally in TPE 2 due to lack of water (Joshi et al., 2007).

CIMMYT target environment characterizations date back to its precursor organization, the joint Mexico-Rockefeller Foundation Office of Special Studies, which operated in the 1940s, and were initially restricted to Mexico but became global with the Center’s formal establishment in the 1960s. Target environment definitions at that time were based on required traits, the need for yield stability, and the diversification of production systems. In the 1970s, 15 agroecological zones were defined. In the 1980s, CIMMYT redefined those agroecological zones and identified instead 12 wheat MEs, defined as broad, not necessarily contiguous but frequently transcontinental, areas with similar biotic and abiotic stresses, cropping system requirements, and consumer preferences (Rajaram et al., 1994); six each for spring and winter wheat growth habits. DeLacy et al. (1993) showed that the major discrimination factors among MEs were latitude and the presence/absence of stresses. Hodson and White (2007) used geospatial criteria to refine ME definitions and found that, in India, ME1 (high yield potential, irrigated) and ME5 (lower yield potential, irrigated, and temperature stressed) were predominant. From our results, and given the geographical projection reported by Hodson and White (2007), TPE 1 overlaps with ME1 and TPE 2 with ME5, whereas TPE 3 relates to ME4 (lower yield potential, drought stressed). The MEs can be broad and transcontinental, making latitude a fundamental factor, but we defined the TPEs in India at the regional level. In fact, when ME1 was first defined in India, it encompassed the NWPZ (TPE 1) and part of the NEPZ (TPE 2), whereas in our analysis, these two regions appear to be different in terms of climate and some soil characteristics (Table 2). Similarly, ME5 included also the CPZ, whereas in our analysis, this appeared to be classified as TPE 3.

The average *H*^2^ in each TPE in all cases was lower than in the SEs. Given that each ESWYT cycle varies from year to year, estimations of GE variance that include not only the SE but also the year effect are not possible due to the lack of connectivity between cycles. Nonetheless, we have somewhat addressed this constraint by analyzing various years of data consisting of “good” and “bad” years in the SEs and TPEs; the year effect is also implied in the pedigree-based prediction models. The average *H*^2^ across sites in India considering TPE subdivision was similar to that of the *H*^2^across SEs owing to the fact that many more environments are present across the TPEs than in the SE, and the response of the genotype within the TPEs becomes G + G^∗^TPE, thus passing this term to the numerator of the *H*^2^ formula and so contributing to the response to selection by separating G^∗^TPE from G^∗^E (Atlin et al., 2000).

The extent of the genotype-by-environment [G^∗^E(TPE)] interaction variance was more than five times greater than that of G across TPEs, whereas across SEs, the G^∗^SE variance was lower than the G variance. Similarly, the average genotypic variance was more than three times greater across SEs than across TPEs. Furthermore, the size of G^∗^E in each TPE was substantially lower when compared to the one across all sites in India [G^∗^E(TPE)], which indicates that the subdivision in TPEs is meaningful by reducing the extent of the genotype-by-environment.

Additionally, the fact that the variance of G increases substantially within each TPE when compared with pooled variance across all sites in India indicates that there is a part of G that can be further exploited in each TPE. Altogether, these results suggest that greater gains can be achieved by targeting selection in the TPE than without TPE subdivision because of a higher precision given that *H*^2^ disregarding the TPEs is substantially lower than the one including the TPEs in the mixed model. Although the size of the G^∗^TPE variance is smaller than that of the G^∗^E(TPE), the size of G^∗^E(TPE) relative to G is substantial (Atlin et al., 2000).

The observed phenotypic and genetic correlations between TPEs suggest that line performance information from one TPE can be used to make inferences about performance in others. This result is useful in the potential design of testing strategies for TPEs, although our results from the variance components still suggest that response to selection can be greater if TPE-targeted decisions are made from the data derived across SEs.

Regarding the CR, the results indicated that with TPE subdivision, the efficiency can be higher in TPE 1 and TPE 2 than across India. The CR for TPE 3 was comparable to that of the analysis across sites, but not higher. Ideally, for the CR to be at its maximum, the *H*^2^ in the SE needs to be higher than in the TPEs, and the genetic correlation needs to be close to unity as possible; however, these conditions are not necessarily met (Searle, 1965). Genetic correlations are highly influential in the CR because its changes are directly related to the opportunities of achieving an indirect response by selecting in the SE for the TPEs (Cooper and DeLacy, 1994). In our results, the *H*^2^ in the SE is higher than in TPEs, and the genetic correlations are, in average, of medium magnitude, which altogether has permitted to make genetic progress in the TPEs. It is important to note that, when calculating the correlated response to selection (indirect selection efficiency), it is assumed that the selection intensity in the SE is the same as in the TPEs (Falconer, 1952; Searle, 1965) for which the observed values may vary, considering that there is higher selection intensity in the SE, going from an initial 9,000 lines to the 49 lines finally included in each ESWYT.

The results derived from the DR indicate that, in terms of selection intensity (*i*), to achieve a selection response across TPEs and in each of the TPEs similar to the one in the SEs, *i* would need to be proportionally higher in each TPE in relation to the SE. This is largely due to the fact that *H*^2^ in individual TPEs is lower than *H*^2^across SEs, so gains in TPEs can be raised by improving TPE testing conditions, thus increasing *H*^2^. One of the aspects that contribute to maintain high-medium *H*^2^ in the SE and that are standard practices are: the mechanized operations (planting, crop management, and harvest), adequate management of the irrigation water including drip irrigation systems, and a crop rotation strategy to uniformize soil moisture during May–September prior to the wheat-planting season in November.

In applying pedigree-based prediction models to assess the predictive ability of SEs, a large portion of total variance was accounted for by the main environmental effects (Table 5), which in this case considers previous information of SEs and TPEs and the TPE/SE–year combinations. In most cases, environmental variance fell significantly with the inclusion of MVs, which indicates this information contains, as expected, an explainable proportion of the environmental variance and the year effect. The SEs in which the MVs did not contribute to total phenotypic variance were BLHT and B2IR, indicating that the major source of variation within these SEs is given by the spatial features of the environment rather than by year-to-year MV variation. In agreement with previous findings, the inclusion of the GE term in the prediction models and environmental covariables generally tends to increase the prediction accuracy of the models (Jarquín et al., 2014; Pérez-Rodríguez et al., 2015; Cuevas et al., 2017).

Based on the pedigree-based predictions (Figure 3), TPE 1 is mainly associated with SEs B5IR and B2IR, and FDRT. The SEs that displayed higher association with TPE 2 were B2IR, F5IR, and FDRT, while B5IR, B2IR, and BLHT displayed higher association with TPE 3. The patterns we found of correlated, and direct, response to selection are being used to select germplasm included each year in ESWYT, which is targeted to TPE 1 and requires lines of normal maturity, rather than the early maturing lines desirable for TPEs 2 and 3.

Previous findings have indicated that simulated SEs at CIMMYT-Ciudad Obregón correlate with international sites and that this has resulted in GY genetic gains in target regions (Trethowan et al., 2002; Lillemo et al., 2004; Tadesse et al., 2010; Manès et al., 2012; Crespo-Herrera et al., 2017). The possibility of evaluating large numbers of lines for GY over different SEs has created an opportunity to develop and adapt germplasm for regions beyond the breeding site. This study suggests expanding the scope of such opportunities. For instance, thanks to CIMMYT’s collaboration with the Government of India and financial support from the USAID Feed the Future Innovation Lab on Applied Wheat Genomics at Kansas State University, more than 500 wheat lines that undergo Stage 2 evaluation are now routinely tested at three sites, each in a different India TPE, by the Borlaug Institute for South Asia (BISA sites; Table 1).

Earlier testing of lines in TPEs, coupled with SE data, could raise genetic gains in ESWYT germplasm (Sharma et al., 2012; Crespo-Herrera et al., 2017), since more information from the TPEs could be available to make crossing and selection decision of parental lines.

In general, the current breeding strategy justifies the indirect selection in the SE for the TPEs, given the magnitude of *H*^2^, the relative size of the G and G^∗^E variances, the magnitude of DR and its implications on the selection intensity, and the fact that a centralized breeding operation that has for a long term delivered genetic gains to India is more resource efficient. Nonetheless, current advances in statistical methods and selection tools may permit a fine-tuning of the testing strategy, for instance, one possible option is to evaluate Stage 1 lines in all the SEs to make selections for Stage 2 evaluations, which also can be conducted in both the SE and TPE. This change would require a modification in trial designs and allocation of resources, as these are limited in both the SE and TPE. Currently, the ESWYT germplasm is tested in the TPEs after 2 or 3 years of testing at CIMMYT-Ciudad Obregón. Hence, earlier information from the TPE can improve the selection for the final ESWYT germplasm and consequently the potential varieties that can be grown in the TPEs.

Based on our results, greater gains in GY were achieved in TPE 1 (118 kg/ha/year) and TPE 3 (123 kg/ha/year) than in TPE 2 (46 kg/ha/year) over the period we studied. ESWYT lines are bred for optimal environments and show better adaptation in TPE 1 and TPE 3. In TPE 2, yield gains are possibly lower due to a shorter growing season with continuous and terminal heat stress, where the germplasm requires some degree of earliness to cope with heat stress (Mondal et al., 2013). These germplasm requirements limit the adaptation of ESWYT lines intended for optimal conditions where earliness is not required due the crop cycle duration. Another annually distributed CIMMYT trial, the International Heat Tolerant Wheat Yield Trial (HTWYT), should feature lines better adapted to TPE 2. The HTWYT lines are selected based on their performance under heat stress with the additional restriction that their performance is comparable to the main checks for the optimal SE.

Wheat breeding must tackle the yield uncertainties that farmers face due to environmental variations. Golling (2006) estimated an annual benefit in excess of $140 million due to more stable wheat yields—independent of gains in genetic yield potential—as a result of breeding research. Yield stability is paramount to smallholder farmers and becoming more important as the earth warms and rainfall grows more erratic. According to Cooper and DeLacy (1994), to develop an understanding of the GE interactions in TPEs, it is important to characterize such TPEs in terms of the key environmental challenges that may lead to the definition of strategies for exploiting the GE interactions. In this study, we defined three TPEs in India based on meteorological and soil characteristics that overlap with the three main wheat production zones in India. Furthermore, the TPEs are associated with a set of SEs at CIMMYT-Ciudad Obregón that have allowed GY gains in the germplasm delivered to the TPEs and across India. The results described herein contribute to an integral strategy to the efficiency of CIMMYT’s wheat breeding program and further ensure the delivery of genetic gains to target environments.

## Data Availability Statement

The datasets presented in this study can be found in online repositories. The names of the repository/repositories and accession number(s) can be found below: http://orderseed.cimmyt.org/iwin-results.php.

## Author Contributions

All authors listed have made a substantial, direct and intellectual contribution to the work, and approved it for publication.

## Conflict of Interest

The authors declare that the research was conducted in the absence of any commercial or financial relationships that could be construed as a potential conflict of interest.
